# “I Wasn’t Gonna Quit, but by Hook or by Crook I was Gonna Find a Way Through for the Kids”: A Narrative Inquiry, of Mothers and Practitioners, Exploring the Help-seeking of Mothers’ Experiencing Child to Parent Violence

**DOI:** 10.1007/s10896-023-00511-z

**Published:** 2023-03-02

**Authors:** Chye Toole-Anstey, Michelle Townsend, Lynne Keevers

**Affiliations:** 1 School of Health and Society, University of Wollongong, Northfields Avenue, Wollongong, NSW 2522 Australia; 2 School of Psychology, Illawarra Health and Medical Research Institute, University of Wollongong, Wollongong, Australia

**Keywords:** Child-to-parent violence, Help-seeking, Intra-action, Family relations, Feminism, Response-based practice

## Abstract

**Purpose:**

Child to parent violence is a significant concern that has been researched over the last sixty years. However, little is known about the help-seeking pathways of parents experiencing child to parent violence (CPV). Barriers and enablers to disclosing CPV have been explored, and responses to address CPV have been nominally researched. The mapping of a disclosure to a choice of where to get help has not occurred. This study seeks to map help-seeking pathways of mothers and considers these pathways in regards to the relations within families and sociomaterial conditions.

**Method:**

This narrative inquiry utilizes response-based practice and Barad’s concept ‘intra-action’ to examine interviews with mothers (*n* = 11) who experience CPV, and practitioners (*n* = 19) who work with families experiencing CPV.

**Results:**

Five help-seeking pathways of mothers are found in this study. Three themes evident across the pathways are explored including: (1) help-seeking within pre-existing relationships; (2) mothers’ feelings of fear, shame and judgement entangled with help-seeking; and (3) conditions which enable and hinder help-seeking from family.

**Conclusions:**

This study finds sociomaterial conditions such as single motherhood and judgement limit help-seeking possibilities. Further, this study finds help-seeking occurs within pre-existing relationships along with the entanglement of CPV with other issues such as intimate partner violence (IPV) and homelessness. This study demonstrates the benefits of employing a response-based approach alongside ‘intra-action’ within a research and practice context.

## Introduction

A child’s violence towards their parent (CPV) is a complex phenomenon. CPV, also known as adolescent family violence, has various definitions ranging from a specific act, to patterns of behaviors, and more often refers to physical violence (Burck et al., 2019). Holt's (2013) definition of adolescent-to-parent abuse is utilised in this paper to define CPV as “…a pattern of behaviour that uses verbal, financial, physical or emotional means to practice power and control over a parent” (p.2). The term child is used instead of adolescent to capture complexities associated with this violence, such as acts by younger children which may not always be defined as violence (see O’Toole et al., 2020). CPV also encompasses violence towards foster carers and other care arrangements including grandparent care and kinship care (Selwyn & Meakings, 2016; Williams et al., 2017). The effects of CPV on mothers may include stress, depression and anxiety (Fitz-Gibbon et al., 2021). Further effects may include isolation, and taking time off work due to the violence which can also have financial ramifications (Fitz-Gibbon et al., 2021). Mothers experiencing CPV may also disconnect from extended family and friends (Fitz-Gibbon et al., 2021; Holt, 2011).

Prior research reports prevalence rates for CPV. The Simmons et al. (2018) review estimated physical violence by young people towards parents in the community to be between 5 to 21%. Concerningly, when emotional, verbal and psychological violence is included, Simmons et al. (2018) found a prevalence rate of between 33 and 93%. However, it is important to note prevalence data is limited by the samples used (Jiménez-García et al., 2020) and the lack of a universally agreed definition (Simmons et al., 2018). Additional challenges related to prevalence data include: parents may not always seek help for the violence (Peck et al., 2021a); perceptions of sex differences where boys use of violence may be perceived as more serious than violence by girls (Selwyn & Meakings, 2016); and there are limited large scale community samples (Papamichail & Bates, 2019).

### Help-seeking and Responses for CPV

Help-seeking is defined as a disclosure of CPV to obtain assistance. Help-seeking is a three stage process of: (1) recognizing a problem; (2) making a decision to seek help; and (3) deciding from who/where to seek assistance (Liang et al., 2005). Seeking help includes informal supports such as family and friends. Help-seeking also includes formal supports, for example: organizations such as child protection or mental health services; emergency services; and public and private practitioners including social workers.

#### Barriers to Help-seeking

Parents may be reluctant to engage help for CPV (Coogan, 2014). Barriers to disclosing CPV include a fear of: ramifications for the child such as criminalizing the child or causing the child harm (Douglas & Walsh, 2018); violence from the child following disclosure (Pagani et al., 2003); being labelled a “bad parent” (O’Toole et al., 2020, p. 12); being doubted or not believed (Clarke et al., 2017); or being blamed by others (Moulds et al., 2016). The risk of removal of other children in the home (Coogan, 2014) or the child using violence (Clarke et al., 2017), leading to child protection involvement, are further concerns of parents when disclosing CPV. The feelings of powerlessness and hopelessness of parents (Cottrell & Monk, 2004), as well as a parent’s experience of shame and guilt they feel for their child’s violence are identified as barriers for accessing support (Omer & Lebowitz, 2016). A feeling of being unable to control their child may inhibit parents disclosing CPV (Williams et al., 2017), as well as feeling incapable as parents (Paterson et al., 2002).

#### Where Assistance is Sought

A recent systematic review (Toole-Anstey et al., 2021) of interventions for CPV found responses targeted at CPV tended to be located within the criminal justice system. Studies have explored responses from settings other than criminal justice such as child protection (Holt & Retford, 2013), and social services (O’Toole et al., 2020). Parents may present to mental health services for issues such as challenging behaviours, as fear and embarrassment prevents disclosing the behaviour as violence (Coogan, 2014). In contexts of kinship care, this same behaviour is understood to be violence and grandparents must present the violence in such a way that it requires intervention whilst also reassuring practitioners that the child is able to remain in their care (Holt & Birchall, 2021). Messiah and Johnson (2017) found parents in their study reported disclosing CPV to family and friends prior to engaging support from formal services. A first response from family or community which minimizes and devalues the violence may prevent further help-seeking (Edenborough et al., 2008).

### Pathways for Help-seeking

Help-seeking pathways for CPV has been minimally researched. The research thus far tends to sit in two strands. One strand explores barriers to help-seeking, while the second strand examines the usefulness of responses. A research gap sits between these two to understand help-seeking pathways, such as exploring how a parent ends up at a mental health service for CPV. To the authors’ knowledge there are no studies which map the journey a parent undertakes for seeking help nor studies which consider the influence of sociomaterial conditions or relations within families. Sociomateriality connects the human to the material, that is the objects and contexts, positioning these as co-emerging with agency forming and re-forming in this connection (Hultin, 2019). This study seeks to address this research gap by examining the following questions: (1) *what are the help-seeking pathways of mothers seeking help for child to parent violence?* (2) *how does the intra-action within family and sociomaterial conditions influence help-seeking?* This paper begins by exploring the theoretical framework and methods for the study. The findings are then outlined, followed by the discussion which incorporates limitations and implications for research, education and policy.

## Theoretical Framework

This research is situated within an emancipationist paradigm (Lather, 2006) and draws on response-based practice (Richardson et al., 2017), and Barad’s (2007) neologism ‘intra-action’. Barad (2007) proposes objects are not independent entities, rather they instead emphasize ‘intra-actions’ between humans and other-than-human relations. Other-than-humans incorporates non-human species such as family pets. This entanglement suggests people ‘become with’ (Haraway, 2008) other-than-human relations, and materialize through the entangled intra-actions. Intra-action shifts thinking from blaming one human for an intentional act, to instead that of practices, relations and sociomaterial conditions creating the possibility for a person to be and act (Webb, 2021) altering the view of CPV. Rather than seeing the parent and child as independent in CPV, this study uses intra-action to view CPV as made up of an entanglement of intra-acting agencies (Barad, 2007; Webb, 2021). Intra-action further moves the considerations of factors which hinder or enable help-seeking, to that of entanglements which make help-seeking for CPV possible and impossible.

Response-based practice is an approach utilized in professions such as social work and sits in the theoretical frame of this research. Response-based practice is a way of viewing violence, whereby resistance is seen as always-already present. This study posits help-seeking is one act of resistance. Response-based practice alters the binaries of victim and perpetrator through elucidating the sociomaterial conditions (such as policy settings, environment and context) in which the social interaction occurs (Richardson et al., 2017). Response-based practice aligns with ‘intra-action’ as both shift the dichotomies of ‘victim’ and ‘perpetrator’ through elucidating other considerations, including context, other-than-humans and sociomaterial conditions in which the violence occurs (Barad, 2007; Richardson et al., 2017). Incorporating intra-action moves the idea of social conditions in response-based practice to the sociomaterial, whereby these ‘factors’ are considered entangled with actions, agency and power (Fomiatti et al., 2022). The novel theoretical framework guiding this research supports the aims of mapping help-seeking pathways to be realized, as well the intra-actions within families and the sociomaterial to be identified.

## Method

A Narrative Inquiry methodology (Wells, 2011) incorporating participatory approaches (Reason & Bradbury, 2006) was utilized for this study. The research, through this theoretical framework and methodology, positions the researcher as part of the world they are seeking to understand (Lather, 2006). The first author is a mother and social worker, and chose to study CPV after working with families experiencing this type of violence. This research is part of a larger study examining parents’ stories as well as responses to address CPV.

### Participant Recruitment

Participants, both mothers and practitioners, were approached through organizations which work with CPV. Organizations approached included violence networks, mental health services, and private social work practitioners. Recruitment of mothers through organizations was intended to support mothers such that if an issue arose in the interview, the researcher would then offer to engage the mother with the worker who provided them information about the study or other services as appropriate. The selection criteria for mothers were (1) child was under the age of 18 at the time of using violence and (2) parent’s self-defined the violence as CPV, and their definition was explored in the interview. The criteria for practitioners was experience working with CPV. This experience may have been working with parents, children or both. Mothers were offered the choice of pseudonym for the research, and all practitioners were assigned a pseudonym. The location of the interview as well as day and time was selected by the participants (World Health Organization, 2016) and a retail voucher was provided to mothers for participation. Questions were asked of mothers via the phone or zoom to support parent safety such as “is now a good time to talk?” (World Health Organization, 2016). In line with response-based practice, mothers were recognized as having capacity to make decisions regarding their safety including their choice to proceed with the interview remotely and discuss the violence. All mothers recruited for the study had engaged support services for themselves and their child in relation to the violence, and as such discussing the violence was not new. Mothers were phoned the next day to check if additional support was required following the interview, similar to other CPV research (Oliver & Fenge, 2020). The study has ethics approval from the University of Wollongong Human Research Ethics Committee.

### Data Collection

Semi-structured interviews (Serry & Liamputtong, 2017) and sense-making co-analysis group discussions were employed for data collection. Interviews were utilized to collect data from parents who have experienced or are experiencing CPV, and practitioners who have worked with CPV. Semi-structured narrative interviews (Anderson & Kirkpatrick, 2016) occurred with mothers face to face, over the phone and on Zoom. Interviews with mothers ranged in length from 40 to 65 min, with an average time of 63 min. Semi-structured interviews were conducted with practitioners face to face, phone and on Zoom. Practitioner interviews varied in length from 53 to 72 min, with 61 min the average time. Mother and practitioner interviews occurred between December 2020 and October 2021.

Two sense-making co-analysis group discussions, herein called co-analysis discussions, were offered to mothers and practitioners. The co-analysis discussions incorporated collective storying of participatory narrative inquiry (Kurtz, 2014), interpretive focus group methods (Dodson et al., 2007), and sequential interviews (Lather, 2001). These discussions were facilitated by the first author: to build meaning of the data (Lather, 2001); further interpret the data (Dodson et al., 2007); increase collaboration with participants (Lather, 2001); and support the emancipatory aims of the project (Lather, 2001). In line with collective storying (Kurtz, 2014), the co-analysis discussions were conducted with people who had already participated in the interviews. The co-analysis discussions were conducted in April 2022.

Interviews and co-analysis discussions were transcribed verbatim, and transcriptions checked with audio recording. Mothers and practitioners were sent their transcripts for member checking with additional questions (Lather, 2001). Participants responded to these additional questions through emails and phone calls, which were recorded and transcribed.

### Data Analysis

Response-based contextual analysis (Richardson et al., 2017) and intra-action (Barad, 2007) were utilized as sensitizing concepts for the data analysis. Response-based contextual analysis incorporates questions such as “what is happening and under what conditions could such interactions occur?… Who is doing what to whom? And how is the victim responding?” (Richardson et al., 2017, p.242). Whilst this study avoids the use of the word victim, these questions support the use of investigating sociomaterial conditions. Similarly, intra-action (Barad, 2007) was utilized as a sensitizing concept to draw attention to entanglements, to understand what goes on between people rather than analytic attention to individuals. Intra-action encourages a focus on CPV in context and in relations rather than on CPV as a ‘thing’ in itself.

The first step for data analysis was mapping help-seeking pathways. Each mother’s transcript was reviewed to map pathways schematically, utilizing the three-stage process of help-seeking as a framework (Liang et al., 2005). A reflexive thematic analysis (Braun & Clarke, 2020) was utilized as the next step. The first author initially familiarized themselves with the data of mothers and coded using the words from the texts (Hesse-Biber, 2010). This coding process was then completed for the practitioner transcripts. The two sets of codes were then developed into themes collectively. These preliminary themes and mother pathways were discussed with the co-authors and refined. This early analysis was considered for cohesiveness with stories from mothers, in line with narrative inquiry methodology (Wells, 2011). The preliminary analysis, with additional questions, was then presented to the co-analysis discussions for further interpretation. The themes and pathways were then refined and re-worked incorporating feedback from the co-analysis discussions. Throughout this process, researcher reflexivity was employed (Braun & Clarke, 2020; Wells, 2011).

## Findings

Mothers (*n* = 11) and practitioners (*n* = 19) from across rural (*n* = 14) and metropolitan (*n* = 16) areas of Australia were interviewed. Practitioners identified as female (*n* = 15) and male (*n* = 4). Although parents were invited, all parent participants identified as mothers and thus the pathways are explored as pathways of mothers. Fields of practice represented by practitioners include homelessness, family and children, mental health, private practice, and women’s services. Practitioners’ professional backgrounds include social work, psychology, family counselling and community work. Children using violence were identified by their mothers as male (*n* = 8) and female (*n* = 6) with an age range of eight to eighteen. Eight of the mothers identified as having experienced IPV from a previous partner. Of these participants, eight practitioners participated across two co-analysis discussions (*n* = 2; *n* = 6).

Mothers report accessing assistance from a range of two to ten informal and formal supports, with an average of six supports for each mother. Formal supports were most common, with 54 formal supports discussed across the interviews with mothers. It is noted that not all assistance directly related to CPV, however 36 supports were discussed in relation to CPV. No differences were found for the number of supports sought for rural and metropolitan mothers.

**Fig. 1:**
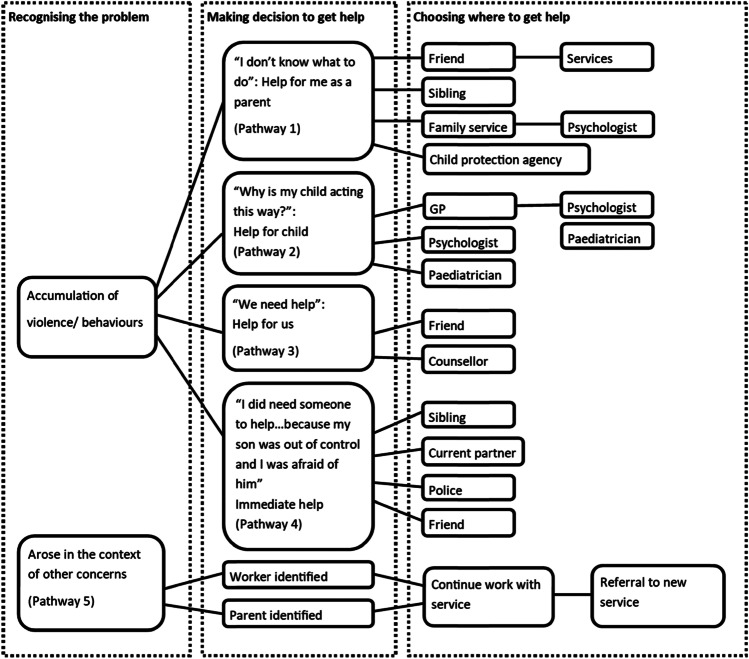
Help-seeking pathways for CPV

The findings are separated into two sections. Firstly, the five help-seeking pathways for CPV found in this study are identified (see Fig. 1) and explained. Secondly, the themes across help-seeking pathways are discussed. The themes are (1) help-seeking occurs within pre-existing relationships (2) mothers’ feelings of fear, shame and judgement entangled with help-seeking and (3) conditions which enable and hinder seeking help from family.

### Help-seeking Pathways

Practitioners indicate more mothers sought help for CPV than fathers. At the co-analysis discussion practitioners agree the presented pathways (see Fig. 1) describe parents accessing their services for CPV. Practitioners from different fields identify some pathways are more representative of their experience than others. Pathways one, two and five were highlighted by practitioners as the most common pathways in the co-analysis discussion. The pathways are not as linear as the mapping illustrates. Some mothers traverse multiple pathways, and other mothers receive referrals to additional services once engaged with a formal service upon seeking help, which is not captured in these pathways.

The three stages of help-seeking (Liang et al., 2005) are discussed for each pathway. Firstly, stage one of recognizing the problem for all pathways is discussed. Each pathway is then analyzed, including the intra-actions within family, sociomaterial conditions and where help is sought.

#### Recognizing the Problem

Mothers indicate recognizing the problem (Liang et al., 2005) of CPV is a two-fold process. Firstly, it involves exploring options and resources they have within themselves. Secondly, once these internal resources are exhausted and if the violence is still occurring, then the problem requires outside (both formal and informal) help. Wendy, a mother, indicates this two-step problem recognition, *“I felt like I’ve run out of options and resources and I really felt for quite some time I don’t know what to do”.* Practitioners also indicate parents exhausting their own internal resources prior to engaging help and highlight, at times, this explains why it may take multiple attempts before a mother accepts help. Stephanie, a practitioner, discusses how some mothers exhaust their options internally and engage multiple times prior to accepting assistance, *“When I spoke to her in the most recent event… she told me that she’s so exhausted and she normally refuses help from anyone and doesn’t need support, but she said that she’s at a wits’ end and she doesn’t know what else to do”*.

Recognizing the problem for pathways one, two, three and four involves *“the re-occurrence of behaviors”* as Linda, a mother, describes. This re-occurrence also includes the challenges with the violence increasing. Wendy, a mother, described the increasing challenges, *“got to the point where the threat of violence was sort of escalating”*. The violent behaviors being recognized as a problem for some mothers included the violence occurring outside of the home. Stephanie, a practitioner, details this escalation *“So, I’ve got a lot of parents that… will tell me they can’t take their child out… because they’re mortified about the potential for the child to be physically violent towards them where other people might see it”*.

Recognizing the problem of CPV in pathway five is different to the first four pathways. The problem of CPV in pathway five is not separate to other issues for which the mother is currently seeking help. Experiences such as IPV and homelessness are part of the help-seeking for CPV in this pathway. There is a tendency here to attribute an artificial cut to the help-seeking, to separate out the help-seeking for CPV as a discrete process. However, in the stories of mothers this help-seeking is not separate, rather the intra-action of CPV with other issues provides the possibility for help-seeking for CPV to become with the other issues. Practitioners, within the co-analysis discussion, highlight other organizations may prompt pathway five such as schools identifying problematic behavior in the classroom and through this process violence by the child in the home is identified.

#### Pathway One: “I don’t Know What to Do”

Pathway one frames a decision to access help for the mother. Parenting is positioned to address CPV in this pathway, implying mothers blame themselves for the violence as well as hold responsibility for resolving the violence. Clare, a mother, explains this responsibility, *“well, I was convinced that I was the world’s worst parent because I can’t control my kids. I can’t alter their behaviors”*. The entangled agency of mothers with their children’s actions is seen here, whereby a mother assumes responsibility for their child’s actions and in turn responsibility for changing these.

Mothers chose both informal and formal services in this pathway including friends, siblings of the mother, family services and statutory child protection agencies. Mothers report informal supports connecting them to formal supports. Donna, a mother, details her friend connecting her with a service, *“the thing is she gets how I feel and what I’m thinking and she’s very quick to try and get – she used to work for [child protection agency], so she’s very quick to try and get services in, like she organized for Susan [practitioner] and all of that”.*

#### Pathway Two: “Why is My Child Acting this Way?”

Pathway two situates CPV as an issue of pathology, with a dualism between the child being responsible for the violence versus the pathology being responsible. This pathway positions the means of addressing CPV is a diagnosis. The diagnosis is not presented as a step, rather a diagnosis explains the violence and this itself addresses the problem. Rachael, from the field of mental health explains this dualism, *“yeah, lots of projecting about, ‘Is my child gonna be a monster? Is there something deeply wrong with them that they’re doing this to me? Why do they have no sense of empathy and no sense of moral compass?’ and not much ability to recognize the dynamic going on between the two”*. This representative quote demonstrates the violence is not viewed relationally, rather it is seen as the responsibility of pathology.

All supports sought in this pathway are medical professionals. Donna, a mother, states *“So that’s why we’re having you know, we’re trying to get all the assessments done to see what’s actually going on”*. This quote illustrates the services in this pathway are selected for the possibility of assessments to diagnose, which will then address the violence.

#### Pathway Three: “We Need Help”

Pathway three is not as represented in the interviews with mothers as the first two pathways. Practitioners in the co-analysis discussions also indicate a paucity of parents accessing their services through this pathway. This pathway frames CPV as entwined within the mother/child relationship and that both mother and child are a part of addressing the violence. One mother, Clare, positions this pathway as one where the help she receives will in turn help her children, *“help me to help them”.* Wendy, another mother, discusses deliberately framing the help-seeking as a “*we*” to ensure she does not attribute blame to her child, *“Can we get some help? We, I never said ‘You, you’re the problem and you need to get fixed’ or anything like that… ‘cause I know that’s never gonna work”.* Supports in this pathway encompass both formal and informal, namely friends and counsellors.

#### Pathway Four: “I did Need Someone to Help because … My Son was Out of Control and I was Afraid of Him”

Pathway four is used when immediate help is required during an incident of violence. All pathways are engaged after the accumulation of behaviors, however pathway four requires help during an incident of violence. This pathway is one which has an immediate safety risk to the mother. The framing of family relations in this pathway situate the mother as needing to be in control. Mothers, through this pathway, indicate supports are needed as *“backup”* for their role as parents which position mothers as agents for addressing the violence.

Practitioners, in the co-analysis discussions, identify parents they’ve seen through pathway four are more likely to have a reluctance or additional barriers to accessing help. Practitioners also attribute a greater risk in this pathway, in part, to a physically larger child. Karen, a practitioner, discusses this larger size of the child, *“I was gonna say often when they’ve gotten bigger than their parents as well, there was always violence even as a child, that’s when it becomes scary”.* Rachel, a practitioner, further discusses the physical size of the child increasing risk,

*“Yeah, that’s right. They’re becoming scary and dad no longer can physically restrain them because they’re scary even for dad now and they’ve been scary for mum for a long time, but now they’re scary for dad and teachers and other people”*

This quote demonstrates the way in which the larger size of the child increases the perception of safety risk for parents, which results in help sought through this pathway. The safety risk here is entangled with the sociomaterial condition of a larger child.

Informal supports such as the mother’s siblings and friends are sought in this pathway. Laura, a mother, states *“It made me feel safe that I could speak to him about it…”* when discussing the safety she feels in calling her siblings for help during an incidence of violence.

Pathway four is the only pathway to involve the criminal justice system, with police called in a time of crisis. Wendy, a mother, explores this option of engaging police,

*“I did need someone to help because I was a woman on my own and my son was out of control and I was afraid of him. I needed that help, but I had no one to call on. The police were the last bastion – when you know – when you’re a woman and you’re that fearful and you’ve got nothing else you can do. Well, I knew that there were cops”.*

The intra-actions of family in this pathway position the mother as needing to be in control, as seen by Wendy’s quote. The intra-actions of family are also considered when discussing the engagement of police by community members witnessing the violence. Stephanie, a practitioner, details how a mother perceived neighbors phoning the police,

*“…her neighbor has phoned the police. And mum’s beside herself because she’s so embarrassed. She’s – ‘Oh, instead of offering to help me with this child that I can’t control, someone’s phoned the police as if I’ve been beating her, as if I’ve been doing the wrong’... So, took… the police intervention the wrong way and just really defensive”*

The mother in this account does not see the neighbor phoning the police as supportive, rather the mother feels it is an indication they perceive her parenting as ineffective.

#### Pathway Five: “I was Dealing with a Younger Sibling who was Presenting with School Refusal …What Panned Out was the Child-to-Parent Violence”

Pathway five is an entangled pathway of help-seeking. The help-seeking for CPV is not separate from help-seeking for other issues. Sometimes parents seek help for other issues such as IPV or homelessness, and the issue of CPV arises after trust and rapport is established with the practitioner. Other contexts in which the issue of CPV may arise, in this pathway, are issues of school refusal, mental health issues or as Karen, a practitioner, indicates *“families facing eviction because of property damage or noise complaints…”* due to the violent (en)actions of a child.

In this pathway, the violence of the child is often bound up with the mothers’ experience of IPV from a male partner. Kathryn describes wanting to respond differently to her son’s violence than she did to the violence from her partner and this is why she sought help, *“I just couldn’t comprehend doing the same with my son”.* This entanglement with a mother’s experience of IPV was also explored in the co-analysis discussions, where Richard, a practitioner, explains,

*“Yeah, some of the hardest things to bear witness to is the crying mother, who says, ‘Here it goes again. I put up with all this crap from their dad, and now my son is doing it’ and that’s that intergenerational cycle and the helplessness and hopelessness that they’re expressing is pretty touching, pretty heavy”.*

This pathway is the only pathway which includes practitioners identifying CPV. Practitioners may witness violence occurring such as during appointments. Leanne, a mother, discusses violent (en)actions by her daughter while residing in a refuge and the way in which the practitioner witnessed and intervened*, “and actually Erica [practitioner] was there and she had a talk to Gabrielle and ended up sending her out, all nice and calm, that really helped.”*

There were mixed findings in the role schools play in identifying CPV. Some practitioners felt schools were missing from this pathway, as Richard, a practitioner, discusses,

*“So pretty much every child does, at some point, attend a school. And certainly in my experience, it’s often where violent behaviors are first seen or starting to be seen which is then fed back to the family, and that could prompt for parents to then start asking these questions of seeking help somewhere”.*

However, other practitioners such as Rachael feel CPV is only within the home *“I think a lot of the time these are behaviors that just happen in the home and that school would have a totally different behaviors”.* No mothers report school being a pathway for help with CPV.

This pathway includes only formal supports. Families may continue to work with services already engaged with for other presenting issues, or in some cases were referred to new services to respond to the issue of CPV.

### Themes Across Help-seeking Pathways

In this section, the themes across the five help-seeking pathways are explored. These themes illustrate sociomaterial conditions which emerge with and in help-seeking for CPV.

#### Help-seeking Occurs within Pre-existing Relationships

Intrinsic to all help-seeking pathways in this study, is a pre-existing relationship with the person. Amy, a mother states *“I rang Susan from [a family and youth service] and ‘cause I…I know her”.* All help-seeking was sought from people with whom family member(s) had previous experience with or a current relationship. Laura, a mother, when travelling pathway two seeking a diagnosis to explain her son’s violence, elucidates the value of the pre-existing relationship to justify why she chose her GP,

*“So I've always – my doctor is awesome. He has a really good connection with Matthew. He's been his doctor since he was born. He does say to me if Matthew ever needs to come for a chat, just to touch base ‘cause he's more than welcome, just to make an appointment, come and have just a general chat about things. But he sees his behavior sometimes, he knows how difficult it is for me at times. So he's a very supportive doctor, which is nice.”*

Practitioners identify a concept of late help-seeking. April, a practitioner states,

*“So, I always find that by the time clients get to me, it’s pretty intense and it’s pretty bad. Like they’ve been everywhere else, and by the time they find myself or other clinicians, it’s completely and utterly out of control… So they’ve usually been putting up with this for a long period of time”.*

This quote is representative of practitioners who feel families attend their service after the violence is entrenched. The finding of help-seeking occurring within pre-existing relationships supports this idea of late help-seeking. Firstly, mothers discussed the internal options they personally used themselves, in an attempt to address the violence prior to engaging external supports. As such, while it may seem help is sought late, in fact they have been actively seeking to address the issue themselves. Secondly, parents may have sought help from other supports within their pre-existing relationships. These relationships may have connected or alerted them to the practitioners whom participated in the research, resulting in these practitioners feeling the help is sought late.

Barriers for seeking help outside of pre-existing relationships are also found in this research. The use of technology to search for services limits possibilities of help-seeking for CPV. Practitioners indicate parents may not know which keywords to use when searching online for services. Rachael, a practitioner in mental health, explains another issue with searching online, *“the amount of psychologists and services that come up is overwhelming, that there’s too many numbers and they don’t know where to go or which one’s the right one”*. April, a practitioner in a rural area, discusses,

“*… this is such a context of being in a rural-remote area, that either they don't think the service exists or the specialists to help them untangle this is so busy that it's such a long wait, that by the time they get to us, it's quite difficult. No one would ever find me in a small rural-remote town by Google, because our anonymity…”*

This quote is symptomatic of the complexities practitioners highlight of using online searching to find services for CPV. Mothers do not report using technology to locate services, and instead relying on their pre-existing relationships for most services in the pathways.

#### Mothers’ Feelings of Fear, Shame and Judgement Entangled with Help-seeking

Mothers’ feelings of fear and shame are entwined with their decision to disclose their child’s violence. Louise, a mother, states when she disclosed CPV to a practitioner she asked, *“…please don’t report this. Please don’t write this in your clinical notes”.* Here, the fear is a concern for consequences for the child using violence and limits the mother’s capacity to talk about the violence she is experiencing. This feeling of fear is also reported by practitioners. Karen, a practitioner, explains some fears mothers have indicated to her such as, *“I don’t want them getting in trouble, or ‘they’d think I’m a dog if I dob them in’ and ‘the families gonna be angry with me’.* Rachael, a practitioner in mental health, also said fear of ramifications include what would happen if mothers were to disclose *“…often [mum] is nervous about telling anybody about it because doesn’t know what’s gonna happen”.* Rachael elaborates, discussing the feelings of fear and shame indicating all families she has worked with experiences this feeling,

*“I don’t think I’ve met a single family where there isn’t fear and shame involved, when there’s child to parent violence involved… There’s usually a heavy level of defensiveness initially, and sometimes outright aggression that other services are suggesting there’s a problem. But underneath that and when you get to know them, there’s a lot of tears and a lot of fear and a lot of shame and that does act as a barrier to help-seeking and they’re often really afraid of what will happen to them as a parent and what will happen to their child”.*

Mothers perceive they will and had experienced judgement when disclosing CPV, limiting ability to disclose the violence. This concern is exacerbated by the sociomaterial condition of being a single mother. Laura, a mother, explains *“I think as a single mum, I feel like people are gonna judge me and say well his behavior’s based on you, which is probably stupid to think that, but that’s how I see it”.* This quote is representative of judgement mothers feel, and limits the possibility of seeking help. Fear and judgement are intensified in rural areas, as Louise, a mother, explains *“they had…no rigorous privacy policies for a small town and safety”* limiting the options available for help-seeking. Tanya, a practitioner working in a small community, similarly discusses this concern in the co-analysis discussion,

*“When there is violent behavior and everything there’s almost a secretive element that I see, so a very big part of ‘why is my child acting this way’, but it wouldn’t be always going to a psychologist or pediatrician, it would be only really talking to friends and family because of the stigma associated to it”.*

Here, the sociomaterial condition of rural areas and small communities co-produce limits to help-seeking for CPV. The supports in a persons’ network is limited by this small community. The material elements, such as formal supports, of the sociomaterial context limit the means through which mothers can seek assistance for CPV.

#### Conditions which Enable and Hinder Seeking Help from Family

Considerations for choosing to disclose CPV to other family members such as parents and siblings of the mother were discussed in interviews with mothers. Mothers, such as Kathryn, identify feeling their parents won’t understand their issues or know how to respond, “*I feel like if I spoke to my mum and dad about it, which I wouldn’t anyway, they just wouldn’t deal with it”.* Other mothers spoke about potential issues this would create in their parents’ relationship with their child, as Linda explains, *“I don’t tell my mum anything because she then takes it out on Morgan”.* Karen, a practitioner, spoke about a challenge parents may experience when disclosing the violence to other family members, *“But I have found quite often that family members can be – kind of reinforce the status quo with the family dynamics. So, sometimes they’ll undermine the mother by blaming the mother as well, like a grandparent”.* Here the perception and experience of a negative social response from the family of mothers, is entangled with help-seeking. This social response limits the options to consider and is implicated in the experience of seeking help.

Mothers, such as Laura, indicate family is a good support for CPV and details her family’s closeness as a reason why, *“She’s a godsend my mum. I’d be lost without her. And we [siblings] all look out for each other, that’s just the way we were brought up”.* The sociomaterial conditions of family in this example, the feeling of closeness together, co-produces the possibility of help-seeking within a family context. The perception of a positive social response enables the possibility to ask for help for CPV.

A notable absence in the help-seeking pathways is the option to engage the child’s father for help. Many of the mothers (*n* = 8) experienced IPV in the past, with some of this enacted by the children’s fathers. The effects of the absence of the father is amplified in the mothers’ experiences of violence, whereby the absence is still influential on the mothers’ relationship with her child and perceptions of gender and safety. Richard, a practitioner, in the co-analysis discussion provides a symptomatic quote of practitioner views in relation to the absent father, *“I’m asking, ‘Where’s the father in all of this?’ If we’re looking at attachment issues, and the first 2000 days, ‘Where’s the father, or the other parent for that matter?’ It takes two to tango to make a baby, where are the two parents or adult role models?’* The sociomaterial condition of fathers who use violence makes it difficult for the mother to engage the father as a support for CPV. Single motherhood in this view, limits the options of pre-existing relationships to draw for support. While other male family members may be available, only three mothers identify approaching males for assistance of which one was a brother, and two were current partners.

## Discussion

This study provides unique contributions to the field of CPV. Firstly, the study identifies sociomaterial conditions and intra-actions bound up with help-seeking for CPV including the contexts for help-seeking and whose family relations responsible for addressing the violence. Secondly, a contribution of this current study is the role of seeking help for other issues such as homelessness in identifying CPV. This section summarizes new knowledge from the study through the frame of the three stages of help seeking (Liang et al., 2005). This discussion incorporates CPV research and help-seeking in the related field of IPV. The methodological considerations of the study, limitations and implications follow.

### Recognizing the Problem

The mothers’threshold’ of violence, that is to recognize when the violent (en)actions of a child are such that outside help is required, is a challenge for CPV. There is an accepted level of violence from teenagers towards parents, such as Holt (2013) states in defining child to parent violence “parent abuse goes beyond the everyday experiences of children ‘hitting out’ at parents, which can happen for all sorts of medical, developmental and situational reasons and is therefore outside the parameters of ‘abusive behavior’” (p.2). This distinction is complicated and implies a social acceptance of violence from children as a ‘normative’ part of development. The point at which a mother perceives the (en)actions of their child as violent as opposed to part of the ‘everyday experience’ is yet to be identified. The threshold of violence in the related field of IPV has been described by women as the point of ‘enough is enough’ (Randell et al., 2011), but there is a lack of consensus of this threshold in CPV. This study concurs with Holt (2013): the lack of understanding the ‘threshold’ of violence impedes recognizing the problem and knowing when to access help.

Mothers experience of both IPV and CPV has been identified in the literature with a recent review terming this “the intergenerational nature of domestic violence” (Peck et al., 2021b, p. 7). Through utilizing the conceptual framework of intra-action (Barad, 2007), this study expands on the connections between these two forms of violence. Intra-action shifts the ‘causal’ connection between experiencing/witnessing IPV in the home and child to parent violence, to that of an entanglement. This entanglement positions the experience of IPV not just as a correlating factor, but a condition which makes the use of child to parent violence possible. The agency of the child in enacting violence is bound up with their experience of IPV. This does not negate responsibility, rather Barad (2007) states an intra-acting lens creates more responsibility and accountability. Further, for some children their exposure to IPV is a condition which renders respectful and non-violent relations with their mother less possible. This is not to imply all CPV is related to IPV, rather for some children their exposure to IPV is a condition which makes the (en)actions of violence possible.

The help-seeking of mothers who had experienced IPV was occasionally entangled, unable to be separated from the experience of violence from their child. This entanglement co-produced the conditions of possibility and impossibility for help-seeking, both limiting and enabling mothers to access supports for CPV. The experiences of the two forms of violence materialized together in seeking help for CPV. Holt (2013) identified mothers who have experienced IPV may be more vulnerable to CPV. This study extends this assertion, finding that the experience of IPV may provide language in which to frame the violence from a child, while this entanglement also produced different avenues of help-seeking.

This current study found, as evidenced in pathway five, CPV is not always the presenting issue. Similarly, Fitz-Gibbon et al. (2021) asserts CPV may not be the presenting problem. Complications in accessing help such as minimal specific early intervention services for CPV, were identified as contributing to not reporting CPV (Fitz-Gibbon et al., 2021). CPV in this study, is found to emerge within the sociomaterial conditions of other presenting issues. The sociomaterial conditions of limited specific services for CPV, as well as not knowing the ‘threshold’ for violence by children, limits the capacity of mothers to seek help for CPV.

### Making the Decision to Get Help

Pathway five captures one sociomaterial condition, residing in a refuge, which makes possible the experience of help-seeking for CPV. Residing in a refuge allows practitioners to witness violent (en)actions of a child towards a mother. “Parenting surveillance” is a phenomenon experienced in refuges, whereby mothers who leave a violent relationship and temporarily reside in a refuge, report scrutiny and regulation applied to their parenting (Fauci & Goodman, 2020, p. 242). A study of mothers residing in domestic violence refuges in the United States found mothers felt their autonomy was restrained and workers in the refuge became involved in parenting (Fauci & Goodman, 2020). In this present study, dissimilar to Fauci and Goodman (2020), the sociomaterial condition of residing in a refuge made it possible for a mother to get support for violent (en)actions of their child.

This study found mothers identified feelings of fear, shame and judgement related to experiences of and perceptions of social responses in help-seeking for CPV. This finding is supported in research of CPV (e.g. Coogan, 2014). However this study, through applying an intra-active and response-based lens, shifts these from factors which hinder help-seeking to entanglements which co-emerge with the experience of CPV. These feelings associated with perceived and real social responses are inseparable from the experience of CPV and are implicated in the help-seeking processes. This implication limits the avenues of where parents can seek help. Similarly, the feelings mothers experience may be limiting the responsibility of the child to change (en)actions. Using intra-action to analyze the feelings experienced shifts viewing these as barriers, to instead co-producing the bounds of help-seeking. The agency of a mother to seek help is limited by these feelings, and is closely tied to the perception of a social response of judgement. Situating mothers feelings through a response-based lens highlights the conditions which makes it possible for mothers to feel blame and shame for the violence of their child (e.g. Williams et al., 2017) and those which do little to preserve dignity or provide recovery for families (Richardson et al., 2017).

Bound up with making the decision to seek help is the intra-actions within family. Pathways one and four placed responsibility with the parents to address the violence. Pathway two indicated a mixed responsibility of the violence with the child and a pathology. The concept of blame on either child and parent and its connection to help-seeking have been nominally investigated (see Holt, 2013). However, an intra-active lens views these family relations as having entangled agencies. These entangled agencies incorporate responsibility to address the violence and influenced where mothers’ sought help. Also implicated in this entanglement is the attribution of how ‘blameworthy’ (Holt, 2013) the mother and child were. These family intra-actions are social conditions which make some pathways of help-seeking possible, with other pathways impossible.

### Choosing Where to Get Help

Research into help-seeking for IPV indicates informal supports are engaged prior to formal services, with one study finding 86.2% of mothers seeking help from family or friends or both (Meyer, 2011). This current study aligns with this finding, and Messiah and Johnson (2017) whom indicate similarly for CPV. This study extends this finding to assert mothers seeking personal and professional help for CPV do so within pre-existing relationships.

The sociomaterial condition of a pre-existing relationship creates the possibility for help-seeking. Extending on the Parentline (2010) survey (cited in Holt, 2013) which indicated GP’s and schools as the main sources of support sought by parents, this study found the condition of a pre-existing relationship as foundational to the possibility of help-seeking. However, schools were not identified by mothers as one of the pre-existing relationships to access for help with CPV in this study. The familiarity and trust in seeking formal support (Holt, 2013) may be additional factors required within the pre-existing relationship. The isolation of parents due to CPV as well as previous/current experiences of IPV may inhibit where parents choose to seek help. The availability of services, as well as confidentiality processes were found to be exacerbated in rural areas in this research, limiting the available pre-existing relationships to draw help from. Rural locations have previously been found to inhibit help-seeking for CPV (Fitz-Gibbon et al., 2021), which this study extends to indicate the sociomaterial condition of a rural location constrains help-seeking for CPV.

Meyer (2011) found some mothers in seeking help for IPV may choose to “remain silent” (p.441) to protect their children for fear of involvement of the child protection system. Similarly, the silence of mothers is documented in CPV (e.g. Burck et al., 2019). This current study found the fear of ramifications for the child, including those related to the child protection system, constrained the decision to disclose CPV, and thereby silence mothers.

The reluctance to engage police for CPV has been found in other research (e.g. Fitz-Gibbon et al., 2021). In previous studies this reluctance to engage police has been attributed to a fear of criminalizing the child (Douglas & Walsh, 2018), not being taken seriously (Edenborough et al., 2008), and not being an effective deterrent (Holt, 2011). This study builds on these findings to consider the relations within family when help-seeking and intra-actions when engaging police. The intra-actions of family relations influenced the pathways mothers’ traversed to seek help for the violence. This current study extends previous studies by finding a condition of engaging police is that a mother must perceive themselves responsible for the actions of a child. Similarly, if mothers bound the agency of a child’s violence with that of pathology a condition of impossibility to engage police was created.

Fathers were absent from help-seeking pathways in this study. Although limited by all the parents interviewed being mothers, the complications of safety for parenting with a father who uses violence towards the mother is present in this study. The conflict of the perceived need for both parents to play a role in child rearing, equally alongside the need to protect children from harm (Simmons, 2018), is present here in these mother’s stories as well as indicated in the practitioner interviews. The isolation of mothers as a sociomaterial condition of IPV (Barnett et al., 2011), such as women leaving their community to escape the violence, is noted in this research. This isolation limits the available options of help-seeking within pre-existing relationships, including from the childs’ father.

#### Methodology Strengths

A strength of this current study is the theoretical framework and methodology. Notably, the inclusion of co-analysis discussions provided depth of analysis in the study. The conceptual frame for the research, utilizing response-based practice and intra-action, provides a structure to elucidate new knowledge. Aligning with Barad (2007) to not provide too much emphasis on ‘words’, utilizing response-based approaches with narrative inquiry, supports the consideration of sociomaterial conditions. The shift of considering previously posed factors as conditions (Webb, 2021), positions the conditions as entangled with agency and power. This positioning provides new insights into help-seeking for CPV. This paper contributes to addressing the gap of ‘silenced mother’s voices’ in research (e.g. Burck et al., 2019). The approach for recruitment in this study addresses this identified gap, and mothers from difficult-to-reach populations were recruited to enable their voices to be present.

### Limitations

The findings of this study should be considered in respect to the limitations. First, the sample size for this research was modest and COVID-19 impacted recruitment. Second, although limited by the cross-over of participants, as practitioners aided the recruitment of parents and thus parents had attended services of practitioners, there was diversity of practice experience drawn on for the research and not all practitioners connected parents with the research. Third, as this study included remote data collection over phone and Zoom, mothers’ may have constrained stories of violence due to other family members being present. Finally, the study is limited by the help-seeking pathways not being temporally mapped.

### Implications

The findings from this study propose a range of implications for practitioners, research and education. First, practitioners should consider their framing of the violence in respect to family relations and responsibility for the violence. Consideration should also be given to parents framing of the violence, in respect to both family relations and responsibility. Practitioners should consider CPV may arise within the context of other issues, may not be the presenting problem when families seek help, and time may be needed to establish a relationship before a disclosure of CPV. Practitioners should be aware of the intra-actions with/in help-seeking for CPV. The heightened risk of violence present in a pathway of seeking help from police needs to be considered by practitioners. Practitioners should consider the sociomaterial conditions which make possible and impossible the help seeking of parents, notably: a rural location or small community; experience of IPV; negative perceptions of social responses; and the need for help-seeking to occur within a pre-existing relationship. Practitioners ought to consider the additional surveillance in refuge settings. Practitioners should acknowledge they are a part of a mothers’ pre-existing network when asked for help, and even if they are unable to deliver a response within service capacity, the support offered at this point may enable or impede further help-seeking.

Second, research is required to identify what role schools may have in identifying and responding to CPV. Further research is required to explore the connection between CPV and other violent behaviors. The ‘threshold’ of violence gap identified in this study requires further research. The implications of practitioner identified CPV warrants examination, as well as if and how this influences the acceptance and willingness of parents to engage in responses for addressing CPV.

Third, training should be provided to alert practitioners to perceptions and experiences of judgement entangled in the help-seeking for CPV. Training should be provided for practitioners to build relationships in the context of help-seeking for CPV. Further research and training is needed to investigate appropriate responses to CPV, which may in turn alleviate feelings of shame, fear and judgement that limits a mothers’ help-seeking for CPV.

## Conclusion

This current study suggests five pathways are traversed by mothers when seeking help for CPV. The analysis points to the pertinence of pre-existing relationships in help-seeking for CPV. The entanglement of CPV with other issues such as IPV, homelessness and school refusal is further elucidated in this study along with concerns of fear, and judgement when seeking help. Incorporating a response-based lens and ‘intra-action’ provides scope for new insights in this current study. Further research to identify the ‘threshold’ for seeking help in CPV is required, alongside the framing of responses from practitioners when parents present to them for help with CPV.
